# An Overview of In Vivo and In Vitro Models for Autosomal Dominant Polycystic Kidney Disease: A Journey from 3D-Cysts to Mini-Pigs

**DOI:** 10.3390/ijms21124537

**Published:** 2020-06-25

**Authors:** Svenja Koslowski, Camille Latapy, Pierrïck Auvray, Marc Blondel, Laurent Meijer

**Affiliations:** 1 Perha Pharmaceuticals & ManRos Therapeutics, Centre de Perharidy, 29680 Roscoff, Bretagne, France; 2 C.RIS Pharma, Parc Technopolitain, Atalante Saint Malo, 35 400 Saint-Malo, France; c.latapy@c-rispharma.com (C.L.); p.auvray@c-rispharma.com (P.A.); 3 Univ Brest, Inserm, EFS, UMR1078, F 29200 Brest, France; marc.blondel@univ-brest.fr; 4 CHRU Brest, Service de Génétique clinique et de Biologie de la reproduction, F 29200 Brest, France

**Keywords:** autosomal dominant polycystic kidney disease, research tool, model organism, biomedical research

## Abstract

Autosomal dominant polycystic kidney disease (ADPKD) is the most common inheritable cause of end stage renal disease and, as of today, only a single moderately effective treatment is available for patients. Even though ADPKD research has made huge progress over the last decades, the precise disease mechanisms remain elusive. However, a wide variety of cellular and animal models have been developed to decipher the pathophysiological mechanisms and related pathways underlying the disease. As none of these models perfectly recapitulates the complexity of the human disease, the aim of this review is to give an overview of the main tools currently available to ADPKD researchers, as well as their main advantages and limitations.

## 1. Introduction

Today, biomedical researchers have a plethora of different tools in hand to study disease mechanisms and investigate potential therapeutic strategies in preclinical drug development. The choice of a specific tool is often the result of a compromise influenced by different factors, not all of them being directed by primary research interests:

1. suitability regarding the specific research hypothesis under study,
2. relevance to the human pathology in terms of causative mechanisms and symptoms,
3. availability and ease of manipulation (e.g., tools already available and well established in the laboratory conducting the research vs. introduction of new technologies),
4. economic factors and rapidness of result obtention.

In preclinical drug development, a combination of different methods is commonly used to identify potential drug targets and therapeutic approaches, beginning often with a cost- and time-effective high-throughput primary screen using in vitro technologies followed by an in vivo evaluation of positive hits in different disease specific animal models (orthogonal screenings and preclinical test). As it is the case for a large number of human pathologies, numerous tools useful for, or specific to, polycystic kidney disease research have been developed over recent decades.

Autosomal dominant polycystic kidney disease (ADPKD) is the most common monogenic inherited kidney disease in humans and the leading inheritable cause of end stage renal disease (ESRD). Its main symptoms are the progressive formation and enlargement of fluid filled renal cysts which gradually impair kidney function until renal replacement therapy, dialysis, or kidney transplantation becomes necessary [1,2,3,4,5]. ADPKD patients present highly enlarged kidneys, which can reach a total volume exceeding 3500 mL for both kidneys combined [6] (versus a mean single kidney volume of 196 mL in healthy individuals [7]). Important kidney enlargement can sometimes be observed even before a significant decline in kidney function. Importantly, ADPKD patients account for about 6% of all patients under renal replacement therapy [8]. In addition, numerous extra-renal manifestations of the disease, such as pancreatic and liver cysts, increased blood pressure, and a higher risk of cerebral aneurisms have been reported [9]. As of today, the selective vasopressin V_2_ receptor antagonist tolvaptan (Jinarc^®^ (EU, UK, Canada), Jynarque^®^ (USA), Samsca^®^ (Japan)) is the only available treatment that can slow cyst growth in ADPKD patients [10,11]. Through the inhibition of the binding of the antidiuretic hormone arginine vasopressin (AVP) to its receptor, tolvaptan diminishes cAMP production and thus cAMP-associated cyst cell proliferation and cyst fluid secretion. However, this treatment is only approved in adult patients at risk or showing evidence of rapidly progressing disease. Furthermore, it is not suitable for all patients due to its risk of hepatotoxicity and some other adverse effects, like polyuria and increased thirst, that can significantly lower the quality of life of people under treatment [12,13].

**Figure 1:**
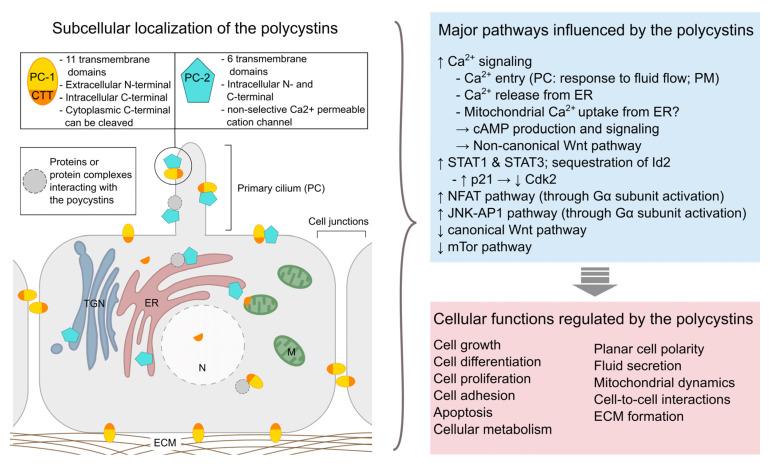
Overview of the subcellular localization of polycystins (**left**) and examples of major pathways and cellular functions influenced by these proteins (**right**). PC-1: polycystin-1; PC-2: polycystin-2; CTT: C-terminal tail (of polycystin-1); ECM: extracellular matrix; ER: endoplasmic reticulum; M: mitochondrion; PC: primary cilium; PM: plasma membrane; TNG: trans-Golgi network. [19,21,22,23,24,25,26].

At the molecular level, ADPKD is caused by mutations in either of two genes, *PKD1* or *PKD2*, which encode respectively polycystin-1 (PC-1) and polycystin-2 (PC-2) [14,15,16]. A third mutated (monoallelic) gene, *DNAJB11*, was recently identified to trigger ADPKD [17]. Mutations in *PKD1* are present in about 85% of ADPKD patients and are related to a more severe disease progression with earlier onset of end stage renal disease [1]. No human case of homozygous *PKD* gene mutations is known and studies in animal models suggest that the *PKD* genes play a crucial role during embryogenesis as homozygous knock-out mice present severe developmental defects and die before birth. Even though the precise pathophysiological mechanisms of ADPKD still remain elusive, extensive research has offered valuable insights regarding the function of the polycystin proteins (Figure 1, for review see [18]). Hence, PC-1 and PC-2 have been shown to interact at the primary cilium of renal epithelia cells, where they form a mechanosensitive cation channel, and ciliary defects appear to be implicated in ADPKD pathology. However, the polycystin proteins are also found in other subcellular locations and seem to be implicated in numerous cell signaling pathways regulating gene expression, cellular differentiation and proliferation, as well as apoptosis [19,20]. 

**Figure 2:**
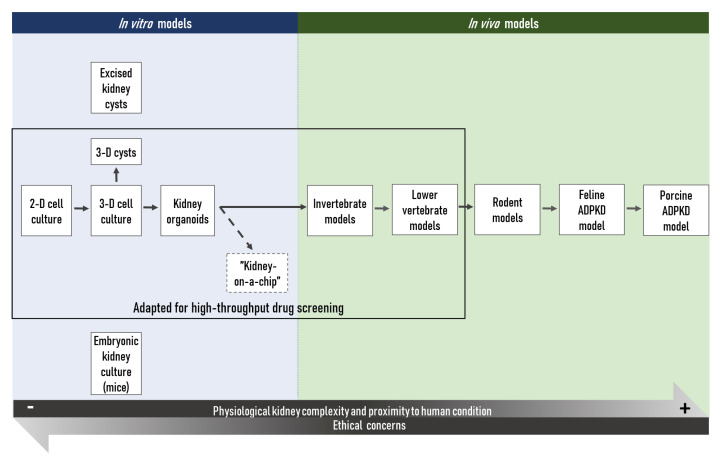
Schematic overview on the different ADPKD research models available to the scientific community. 2D: two-dimensional; 3D: three-dimensional.

The level of functional polycystin proteins produced in the cell seems to be a crucial factor for renal cyst formation and decreased expression of the *PKD* genes can lead to ADPKD development in humans as well as animal models of the disease [27,28]. Importantly, renal injury seems to accelerate disease progression through the activation of repair mechanisms [29,30,31]. However, much about the pathogenesis of ADPKD remains to be learned and the aim of this review is to give an overview of the most important in vitro and in vivo tools available for ADPKD research, some of the most relevant progress achieved through the use of those tools as well as an update on the emerging technologies (Figure 2). Hence, this article should hopefully help researchers to choose the best suited models for their specific studies and possibly to design the most efficient approaches to investigate their hypotheses.

## 2. In Vitro Research Tools

Numerous in vitro models have been developed in order to unravel ADPKD disease mechanisms and carry out drug screenings with the aim to identify potential therapies for ADPKD. Those models have evolved from simple monolayer cultures of diverse kidney epithelia cell lines to 3Dimensional (3D) cystic structures and then to PKD (polycystic kidney disease) specific kidney organoids. While these models clearly represent valuable tools to unravel cellular mechanisms and genetic interactions relevant to ADPKD, as well as to carry out initial large-scale drug screens, one needs to bear in mind that these models are not representative of whole organism physiology, notably due to the absence of their natural environment. Thus, even though considerable progress is made to develop more representative in vitro models, when interpreting results of such essays researchers have to consider that the cellular models used today are lacking important aspects, such as tissue interactions or complex drug metabolization. Therefore in vivo validation of results is still a mandatory step for understanding ADPKD pathophysiological mechanisms and drug development. Furthermore, and concerning specifically ADPKD research, renal cell culture systems, or even kidney organoids can only mimic the nephrological aspects of the pathology. Even if this aspect is predominant in the disease, one wants to keep in mind that rather frequent extrarenal manifestations of the disease—such as vascular defects—exist that cannot be taken into account in these various models. A broad overview of the main (general and ADPKD-specific) advantages and limitations of the different in vitro tools is presented in Table 1. For more detailed information about the different in vitro tools the readers are referred to the references used to establish the Table 1, in particular to a recently published book chapter describing the applications of kidney organoids for PKD research and containing several detailed protocols by Jinghua Hu and Yong Yu [32] and to an excellent review on human cell-based models used in ADPKD research published by Weydert et al. in 2019 [33].

### 2.1. Cultured Monolayers of Kidney Cells and Three-Dimensional Cysts

Diverse immortalized kidney cell lines have been used to study ADPKD disease mechanisms and carry out drug screening assays. The Madin–Darby canine kidney (MDCK) cell line was isolated by Madin and Darby in 1958 from an adult female cocker spaniel [42,43]. This cell line retains differentiated kidney properties. When cultured on a solid surface, MDCK cells form a continuous and fully polarized epithelial sheet and vesicles appear, the formation of these vesicles is due to fluid transport in an apical-to-basolateral direction where the accumulation of fluid lifts the cell sheet away from the culture dish [44]. In 1987, McAteer et al. described a method permitting to initiate renal epithelial cyst formation with MDCK cells when cultured in a three-dimensional (3D) collagen matrix [45]. The fluid-filled cysts formed by these cultured MDCK cells have the basolateral surface directed towards the collagen matrix, fluid flow thus occurs in the opposite direction from the flow responsible for the formation of vesicles when the cells are cultured on a two-dimensional (2D) surface and therefore better mimics fluid transport in cysts of ADPKD patients [46,47]. In 1989, Mangoo-Karim et al. showed that renal epithelial cells of a different origin, especially normal human kidney cells and cells extracted from ADPKD kidneys, are also able to form fluid-filled cysts when cultured in a 3D collagen matrix [48]. Diverse renal epithelial cell lines in 2D or 3D culture systems, as well as excised cysts from ADPKD kidneys have been extensively used since the 1980s to study the molecular mechanisms at the basis of cyst formation and growth in ADPKD and to identify potential therapeutic molecules. These studies notably helped to identify dysregulated fluid and solute transports as promoters of cyst formation and growth. They also revealed the crucial role of cAMP in cyst enlargement through its implication in fluid secretion and cell proliferation. In addition, studies using these in vitro tools showed that diverse molecular transporters as well as receptors seem to be abnormally located in ADPKD cyst-lining epithelia, and that diverse signaling pathways are implicated in ADPKD pathogenesis [48,49,50,51,52,53,54,55,56,57,58,59,60,61,62,63,64,65]. A detailed protocol for in vitro cyst formation was recently published by Sharma et al. [47]. Furthermore, a high-throughput screening platform using 3D cultured cysts was described by Booij et al. in 2017 [66]. This platform was developed in order to identify compounds affecting cystogenesis, thus allowing to screen for potential therapeutic molecules which prevent or slow down cyst formation, and to unravel new molecular pathways involved in cyst swelling. 

In addition to normal kidney cells, any epithelial kidney cell line isolated from transgenic or non-transgenic ADPKD animal models can be used to study the cellular mechanisms of ADPKD pathology. As an example, Felekkis et al. used, among other cell lines, primary tubular epithelial cells from a transgenic PKD rat model expressing a truncated polycystin-2 protein, to investigate p57/Cdk2 contribution to increased cell proliferation observed in ADPKD cyst lining epithelial cells [67]. Finally, a very appealing high-throughput approach for ADPKD drug screening has very recently been published by Asawa et al. [68]. In this paper, a comprehensive screening of a large compound library (~8000 compounds) is described. It is based on high throughput viability and 3D cyst growth assays performed on primary human renal cells and immortalized murine cell lines, comparing, for each compound, activity between *Pkd1* deficient and wild-type cells in order to identify molecules which preferentially affect *Pkd1-*mutant cells. The initial screening approach was carried out based on a viability assay performed on *Pkd1*-null (MEK-null) and wild-type (MEK-wt) murine embryonic kidney collecting duct cells, as well as *Pkd1* null (PN24) and *Pkd1* heterozygous (PH2) murine adult proximal tubule cells. While only a minority (~18%) of active compounds presented differential activity in both murine cell pairs (*Pkd1* deficient vs. wild-type), a vast majority presented differential activity in embryonic but not postnatal kidney cells, thus underlining the importance of the origin and initial developmental stage of cell lines. Then, the compound’s efficiency in cyst reduction was evaluated thanks to a 3D high throughput cyst swelling assay based on PN24 cells (no cyst formation could be reached with MEK cells). Finally, in order to evaluate whether the identified compounds were relevant to the human disease, a viability assay, comparing primary normal human kidney (NHK) and cyst-lining cells from ADPKD patients (unfortunately, no information on cyst origin and mutation type was provided) was used and identified 21 molecules, among those some compounds that have previously been evaluated in the context of ADPKD pathogenesis, which further validates this approach. In addition, this study underlines the importance of the choice of the cell lines used for such an assay, as different results can be obtained when using cells originating from two distinct albeit similar murine ADPKD models, and that a validation of the results with cells of human origin is mandatory. The variability that was observed by Asawa et al. with ADPKD cells of human origin further underlines the importance of the specific genetic background of the cells. Hence, detailed information about their exact origin and the specific ADPKD mutation present should be gathered, if possible.

### 2.2. Embryonic Kidney Culture

Metanephric organ culture of mouse embryonic kidneys in vitro has been developed to study kidney organogenesis [69]. Such wild-type or transgenic mice embryonic kidney cultures have been used to study mechanisms of cyst formation and decipher the implicated molecular pathways. These studies helped to reveal the implication of CFTR (cystic fibrosis transmembrane conductance regulator), a cAMP regulated chloride channel, in cyst enlargement. Indeed, in 2008, Yang et al. showed that CFTR inhibitors are capable of slowing cyst formation in vitro. They used embryonic kidney cultures of wild-type mice in which 8-Br-cAMP was employed to initiate cyst-formation combined with an in vivo approach based on a *Pkd1* kidney-specific knock-out strain [70]. Magenheimer et al. used embryonic kidney cultures from wild-type, *Cftr* knock-out, *Pkd1* knock-out or combined *Cftr* and *Pkd1* knock-out mice and demonstrated that *Cftr* knock-out completely suppressed 8-Br-cAMP- as well as *Pkd1* knock-out-induced cyst formation [71]. The role of aquaporin-1 in retarding cyst growth was demonstrated by Wang et al. in 2015 [72]. They designed an elegant study combining in vitro and in vivo approaches, among which the culture of embryonic kidneys from aquaporin-1 null mice as well as from aquaporin-1 expressing mice after induction of cyst formation by 8-Br-cAMP. 

### 2.3. Stem Cell Approaches and Kidney Organoids 

As stated by De Souza, “an organoid is a 3D multicellular in vitro tissue construct that mimics its corresponding in vivo organ, such that it can be used to study aspects of that organ in the tissue culture dish” [73]. The development of organoids has been made possible thanks to the observation that stem cells (pluripotent embryonic or induced stem cells and adult stem cells) are able to assemble into complex tissue-like structures under specific culture conditions. This means providing appropriate differentiation signals in the case of embryonic stem cells (ESC) and induced pluripotent stem cells (iPSC), or providing signals inducing tissue repair or maintenance in the case of adult stem cells. In a 2013 publication, Takasato et al. described the successful generation of kidney organoids from human embryonic stem cells (hESC) [74]. Since then, protocols for kidney organoid production from stem cells have been refined and several methods have been developed and published, thus revealing ever more potential medical and research applications for this technology (for review see [75,76]).

In 2011, Thatava et al. described for the first time the generation of iPSC starting from cells originating from an ADPKD kidney of a patient carrying the W3842X mutation in exon 41 of the *PKD1* gene [77]. The team of Benjamin S. Freedman at the University of Washington School of Medicine is specialized in the use of iPSC and CRIPSR/Cas9 technology in order to model human kidney disease with the aim to develop “clinical trials in a dish” [78]. In 2013, Freedman et al. used iPS cells derived from ADPKD kidneys in order to study whether the level of ciliary polycystin-2 is influenced by a reduced level of functional polycystin-1 [79]. Two years later they demonstrated that kidney organoids derived from iPSC cells can be used to study ADPKD specific pathophysiological mechanisms. They induced a *PKD1* or *PKD2* knock-out in hESC cells using the CRISPR/Cas9 technology and observed tubular cyst formation in derived kidney organoids [80]. In 2017, this team further showed that kidney organoids are capable of modifying their microenvironment and that this ability partly depends on PC-1 [81]. In addition, their results indicate that PKD patient derived iPS cells show a high variability in their capacity to form organoids and that variations in tubular morphology were observed when organoid formation was successful. They concluded that CRISPR-generated mutant hPSC carrying either a *PKD1* or *PKD2* knock-out are, at the current state of technology, more suitable to study PKD specific effects than patient derived iPSC. Finally, using hPSC derived kidney organoids, the Freedman Lab developed the first platform for automated organoid differentiation allowing high-throughput screening (HTS) [36]. In addition, they demonstrated that this HTS system is suitable for disease modeling and drug screening by showing that it produces reproducible results when used with kidney organoids derived from *PKD* knock-out hPS cells. In a study published in 2018, Boreström et al. also reported the development of a refined protocol for kidney organoid generation with high-throughput applicability [37]. By using the CRISPR/Cas9 technology they generated kidney specific reporter lines that permit the monitoring of kidney differentiation, glomerular maturation, and podocyte health in living cells. 

In addition, patient derived iPS cells and kidney organoids are also used to study other specific aspects of ADPKD pathology. In line with this utilization, the differentiation of ADPKD patient derived iPS cells into vascular endothelia cells was used to study cardiovascular complications associated with ADPKD. Using this approach, Ameku et al. revealed altered Ca^2+^ entry and gene expression in ADPKD patients-derived endothelia and were able to identify MMP1 as a potential novel risk factor for intracranial aneurysms in ADPKD patients [82]. Here again, we refer the reader to the recently published chapter describing several protocols and numerous valuable resources for the application of kidney organoids in ADPKD research, in the book *Polycystic Kidney Disease* edited by Jinghua Hu and Yong Yu [32].

Even though kidney organoids are organ-like structures composed of different kidney specific cell lineages, they nevertheless lack crucial characteristics of a complete functional kidney as organoids are not vascularized and not subject to the kidney specific microenvironment and fluid flows present in vivo. In response to this problem, an emerging technology is the development of cell-based microphysiological systems, the so-called organs-on-a-chip [83]. Culturing cells in microchannels and microfluidic systems permits the recapitulation of in vivo tissue- and organ-level functions in an ex vivo system. However, while this technology is very promising for the field of ex vivo drug screening and testing, and while on-a-chip models of isolated functional components of the kidney have successfully been created (for review, see [83]), there is no bona fide kidney-on-a-chip available today. Hopefully, the ongoing research and refinement of protocols for organoid production and organ-on-a-chip technology will finally lead to such a valuable tool in the near future.

## 3. In Vivo Research Models 

A large number of in vivo models are available for the study of ADPKD-specific disease mechanisms and drug testing, the most common being rodent models. Animal models used in ADPKD research either recapitulate human pathology induction through the introduction of mutations in the corresponding *PKD* gene orthologues, or present a phenotype closely mimicking the hallmarks of ADPKD pathology, such as kidney and liver cyst formation and slow disease progression. The knock-out of *PKD* orthologous genes and the observation of a consecutive ADPKD-related phenotype in various animal models can help gain insights into the molecular mechanisms of pathology development. 

Invertebrate or lower vertebrate organisms like *Xenopus* or the zebrafish can also be valuable tools, in particular for high-throughput in vivo drug screening [84,85]. Another advantage of these organisms for biomedical research is that they do not fall under the same strict directives on the protection of animals used for scientific purposes in the European Union, at least until a defined developmental state, which in the case of vertebrates is the independently feeding larva [86]. Whereas invertebrates present a fast generation time, which permits the rapid generation of models and results, the generation time of lower vertebrate such as zebra fish or xenopus is comparable to those of rodent models (sexual maturity is reached 3–4 months post-fertilization in *D. rerio* and at about 4–6 months of age in *Xenopus* frogs) [85,87]. An overview of the most important advantages and limitations of the invertebrate and lower vertebrate models is presented in Table 2. Mammals, however, are genetically and physiologically more similar to humans than the aforementioned organisms, thus studies conducted in mammals might better reflect human ADPKD disease mechanisms and permit more reliable observations, in particular regarding determination of treatment efficiency and adverse effects. However, as compared to invertebrates and lower vertebrate models, higher maintenance costs, less abundant progeny, and slower generation time do not easily permit the use of mammalian models for large scale drug screening, and thus limit their use to the in-depth analysis of efficiency and toxicity of otherwise identified drug candidates and to their preclinical validation before clinical testing.

### 3.1. Invertebrates 

Even though one would not suppose that invertebrates, that do not have a excretory system as complex as the mammalian kidney, could find an application in the study of human chronic kidney disease, research carried out on the nematode *Caenorhabditis elegans* and the fruit fly *Drosophila melanogaster* have helped gain valuable insight in ADPKD disease mechanisms. 

For example, the group of Maureen M. Barr has focused on the study of human disease mechanisms in the *C. elegans* worm model. In their landmark paper from 1999 Maureen M. Barr and Paul Sternberg described the *C. elegans* homologues of PC-1 and PC-2, LOV-1 and PKD-2 respectively, which are expressed in the worm ciliated neurons and locate to cilia, though they are not required for their assembly [88,89]. They further report that both, *lov-1* and *pkd-2* mutations induce impaired mating behavior in *C. elegans* males and suggest that LOV-1 and PKD-2 might act in a common sensory signaling pathway. Since then, *C. elegans* has become a tool to study protein interactions and molecular signaling pathways in the cilium as well as potential disease mechanisms associated with ciliary dysfunction (ciliopathies), including polycystic kidney disease (for review see [90,91,92,93]). Several direct interaction partners or indirect modifiers of LOV-1 and/or PKD-2 expression, localization, and activity have been identified in *C. elegans* and human orthologous genes exist for many of those [94,95,96,97,98,99,100]. Thus, these works based on *C. elegans* contributed important insights into the molecular functions of polycystins and hinted to potential pathophysiological mechanisms and modifier genes for ADPKD. We also want to refer our reader to the recently published chapter describing several protocols and numerous valuable resources for the application of *C. elegans* in ADPKD research, in the book “Polycystic Kidney Disease” edited by Jinghua Hu and Yong Yu [101].

As for the fruit fly *Drosophila melanogaster*, ADPKD research has mainly focused on defects of *Amo*, the fly homologue of *PKD2*. It has been shown, that similarly to *C. elegans*, defects in the polycystin-2 homologue impair fruit fly male fertility. In *D. melanogaster,* Amo is localized at the tip of the sperm flagella, a motile ciliary structure, suggesting a role in signal transduction upon transfer to the female reproductive tract [102,103,104,105]. Furthermore, a *D. melanogaster* model producing an ADPKD phenotype was described in 2017 by Gamberi et al. [106]. They described that a knock-out of the fruit fly translation regulator Bicaudal C (*BicC*) causes progressive cystic degeneration of renal tubules that can be reduced by rapamycin treatment. As *BicC* expression has been found to be downregulated in PKD patients, the authors of the study suggested that *BicC* acts downstream of *PKD1* in a conserved molecular pathway. Furthermore, the C-terminal cleavage product of mouse polycystin-1 was over-expressed in *D. melanogaster*, revealing that PC-1 might be implicated in regulation of the mitochondrial function [107]. 

### 3.2. Lower Vertebrates

Extensive ADPKD research has also been conducted in zebrafish and *Xenopus* models. In *Xenopus*, extensive studies of polycystin protein function and the molecular pathways implicated in polycystin expression, trafficking, and complex formation were performed. Several important properties of the polycystins as Ca^2+^ permeable cation channels [108,109] as well as the capacity of the PC-1 C-terminal cytoplasmic tail to induce Cl^-^ currents in transfected oocytes [110] were revealed through in vivo studies using *Xenopus*. In elegant studies combining several in vitro and in vivo techniques, Streets et al. demonstrated the role of PC-1 moderated PC-2 phosphorylation as a regulator of Ca^2+^ release and cell growth, Xu et al. characterized several binding partners and functions of the highly conserved polycystin-1, lipoxygenase and α-toxin (PLAT) domain, whereas Kim et al. identified several proteins implicated in the Wnt/Ca^2+^ pathway as in vivo and in vitro binding partner of the polycystins [111,112,113]. Furthermore, thanks to the use of specific morpholino-oligos raised against one amongst several genes, a PKD-like phenotype could be induced in *Xenopus*, thereby leading to valuable insights into the signaling network underlying ADPKD pathology. Indeed, the single knock-out of *Xenopus Pkd1* [112,114], *Pkd2* [115], *Bicc* [116], or *Gnas* (Guanine nucleotide-binding protein G subunit α isoforms (short)) [114] induces this PKD-like phenotype that includes edema, due to impaired kidney function, as well as dilated pronephric tubules and ducts. This work also revealed the implication of *Bicc* and the miR-17 micro RNA family in the regulation of *Pkd2* mRNA translation. Furthermore, the interaction between the C-terminal tail of polycystin-1 and G-protein α-subunits was shown to play a critical role for correct kidney function and to be an important inhibitor of aberrant G-protein β/γ signaling. 

In the last few decades, the zebrafish *Danio rerio* has been increasingly used for drug screening and as a relevant and convenient system to model various human kidney diseases (for review see [117,118]). As for ADPKD, this model has led to important insights into the pathways modulated by the polycystin proteins. The C-terminal cytoplasmic tail of polycystin-1 was shown to be implicated in Wnt signaling [119] and in the STAT6/p100 pathway [120] thanks to in vitro experiments and overexpression of the PC-1 C-tail in zebrafish embryos. In addition, several genes implicated in cilia assembly or function have been shown to induce kidney cysts and, interestingly, body curvature (ventral or dorsal) when mutated in zebrafish lines or in specific knock-outs induced via morpholino injection [22,121,122]. Of those models, *Pkd2* and *Pkd1a/b* (the two *Pkd1* isoforms with different expression patterns identified in zebrafish [22]) and *bicC* morpholino-mediated knock-outs seem to be the most relevant for ADPKD research. Indeed, they all develop kidney cysts and dorsal body curvature without any apparent defects in cilia assembly or structure, while other models presenting kidney cysts generally develop ventral body curvature [121]. Furthermore, a comparative study of cyst formation and cilia function between *locke*, *swt*, and *curly* mutants (the three developing renal cysts and ventral body curvature) and *Pkd2* morphants suggests that the mechanisms underlying cyst formation in *Pkd2* morphants differ from those observed in these three mutant strains [123]. Importantly, the phenotypes observed in these zebrafish ADPKD models can most often be rescued by injection of mouse or human mRNA encoding the corresponding protein orthologue [122,124]. Furthermore, *Pkd2* morphants present left-right patterning defects similar to those observed in *Pkd2* knock-out mouse embryos [125,126]. In 2008, Fu et al. used this *Pkd2* morpholino model to demonstrate that polycystin-2 is probably involved in different cellular functions depending on its intracellular localization. Indeed, various and specific phenotypes of the *Pkd2* zebrafish model were rescued by injection of a mutant PC-2 mRNA targeted to various and specific subcellular localizations, supporting the hypothesis that polycystin-2 exerts tissue-specific functions depending on its distinct subcellular localizations [127]. Furthermore, *Pkd1a* morpholino injection induces the development of liver cysts in zebrafish embryos [128]. In 2006, Low et al. made the interesting observation that overexpression of the PC-1 cytoplasmic C-terminal tail in zebrafish leads to kidney cysts in the absence of body curvature [120]. In addition, polycystin-mediated defects in collagen expression during zebrafish embryogenesis could be revealed in a morpholino-induced *Pkd2* knock-out model. The authors suggested that polycystins are implicated in a negative feedback signaling pathway regulating the expression of several collagen genes in response to mature crosslinked extracellular matrix (ECM) formation [22]. Also, in 2014, Sussman et al. identified two zebrafish homologues of the phosphodiesterase 1A (PDE1A), an enzyme interesting in the context of ADPKD because of its capacity to hydrolyze cAMP and as the unique PDE isoform regulated by Ca^2+^ [129]. They demonstrated that PC-2 and PDE1A might function in a common pathway and that ADPKD pathology might be modulated via PDE inhibition. Here again, we want to refer our reader to the recently published chapter describing several protocols and numerous valuable resources for the application of *D. rerio* in ADPKD research, in the book “Polycystic Kidney Disease” by Jinghua Hu and Yong Yu [130].

### 3.3. Drug Screening Approaches in Invertebrate and Lower Vertebrate Models of ADPKD

Invertebrate and lower vertebrate models present a number of advantages that make them attractive for drug screening approaches (Table 2). These advantages include notably the relatively fast and easy construction of transgenic animals and the numerous progeny that allow to produce a large number of (transgenic) animals in a short time frame. In addition, the ex utero development and very small size (at least at some developmental stages) allow to perform high throughput screening assays relatively easily.

Nevertheless, as of today, and to the best of our knowledge, no ADPKD specific drug screening assays have been carried out in *C. elegans* or *Xenopus* models of ADPKD. In contrast, a handful of studies have employed *Drosophila melanogaster* models to carry out drug screening assays or to study effects of specific drugs. Hofherr et al. studied the effect of a patient specific *PKD2* mutation (*PKD2*^D511V^) and described the use of *D. melanogaster* as a readout for polycystin-2 channel function in vivo [104]. Following in vitro assays with cellular models, they suggest that the *PKD2*^D511V^ mutation compromises PC-2 channel folding, thus promoting lysosomal degradation. In accordance with this, treatment with the FDA-approved lysosomal inhibitor chloroquine was able to increase protein levels of the mutant PC-2 channel in vitro. In order to carry out an in vivo validation of these results, the patient specific mutation (*Amo*^D627V^, equivalent to human *PKD2*^D511V^) was introduced in *Amo* deficient male flies (*Amo*^-/-^). The double transgenic animals (*Amo*^-/-^; *Amo*^D627V^) showed slightly increased fertility compared to the *Amo* deficient male flies, indicating that the mutated protein presents some residual function in vivo. Administration of chloroquine in the food led to a partial rescue of the reduced fertility only in the double transgenic animals, which correlated with an increased expression of the mutant protein in sperm flagella of chloroquine-treated *Amo*^-/-^;*Amo*^D627V^ flies. This study thus demonstrates that thanks to the time- and cost-effective production of transgenic animals, *D. melanogaster* could represent a valuable model to study the effects of patient specific *PKD2* mutations in vivo and to develop screening assays that aim at identifying compounds specifically suppressing the effect of these mutations. This system is however limited by the absence of a *PKD1* homologue in the fly, thus permitting only to evaluate *PKD2* specific mutations. In addition, interactions between the polycystin proteins, which are observed in humans and other organisms and are implicated in the regulation of their function and trafficking to specific subcellular localizations can therefore not be studied in this model. This represents a major limitation as defects of protein trafficking are thought to be implicated in ADPKD pathogenesis. In 2019, Millet-Boureima et al. tested the effect of Smac (second mitochondria-derived activator of caspases) mimics, of which one was previously shown to suppress the pathological phenotype in an ADPKD rat model, in the *BicC* mutant fly model of ADPKD and found important cyst reduction upon Smac treatment [134]. Hence, the *BicC* model might be useful to conduct high throughput drug screening aimed at isolating potential therapeutic molecules in ADPKD. However, as *BicC* is thought to act downstream of *PKD1* in ADPKD patients, and as a *PKD1* homologue has not been identified in *D. melanogaster*, potential differences in the signaling pathways implicated in the development of the renal cystic phenotype between human ADPKD patients and the *BicC* fly model might limit its relevance, both as a model for the human disease and for drugs screening.

The vast majority of drug screening studies using invertebrate or lower vertebrate models of ADPKD employed the zebrafish *D. rerio*. Cao et al. identified histone deacetylase (HDAC) inhibitors as candidates for PKD treatment using zebrafish *Pkd2*/hi4166 (which does not develop a cystic phenotype but instead a dorsal body curvature) and *ift172/hi2211* mutants (developing ventral body curvature and kidney cyst formation), zebrafish *Pkd2* morphants and a murine kidney specific *Pkd1* knock-out model [140]. In 2016, Wu et al. employed a multimodal approach, using a 3D cyst assay with human renal epithelial cells (immortalized and normal), zebrafish *Pkd2* morphants and the Han:SPRD rodent model, which led to the identification of a beneficial effect of resveratrol on PKD progression, as well as of NF-κb as a potential therapeutic target [141]. Furthermore, Chang et al. demonstrated that metformin—which has previously been shown to reduce cyst growth and fluid secretion in 3D cell culture assays, murine embryonic kidney cultures, and rodent models of ADPKD—also reduces pronephric cyst formation and dorsal body curvature in zebrafish *Pkd2* morphants [142]. The dorsal axis curvature is often used as an indicator of efficiency as its severity is dose-dependent (morpholino injection) and, in contrast to the presence of cysts, it is the most penetrant and consistent feature observed in zebrafish ADPKD models [22]. An automated imaging pipeline in 96-well plates using larvae of zebrafish in which the developing pronephros is highlighted by GFP (Tg(wt1b:EGFP)) has been developed by Westhoff et al. and validated for nephrotoxicity screening. They further demonstrated that this system can be used to detect phenotypic abnormalities (cyst development and ventral curvature) caused by morpholino-induced *ift80* and *ift172* knock-out [143]. In future studies, this system could thus potentially be employed for drug screening assays with *Pkd* specific morphant embryos. In addition, a recent study identified ALK5 kinase (or TGFßR1 - transforming growth factor, beta receptor I) and non-canonical androgen receptors as potential targets for ADPKD drug development using a high throughput screening approach based on a zebrafish *Pkd2* mutant (using tail curvature as a primary readout) followed by a secondary validation in 3D cyst assays based on MDCKII and a human *PKD1* patient-derived cystic cell line (OX161c1) [138]. However, as significant phenotypic variability has been described between zebrafish *Pkd2* morphants models and/or *Pkd2* mutant models, standardized protocols should be developed to ensure comparability and reproducibility of the results when carrying out drug screening assays based on this model. Finally, zebrafish contains two *Pkd1* paralogs with distinct expression patterns and the single knock-out of *Pkd1a*, or the simultaneous knock-out of *Pkd1a* and *b*, induces kidney cysts only in a small fraction of embryos (not exceeding 20%) and even though signs of kidney failure like severe edema are observed more frequently, a hydrocephalus and jaw defects seem to be the primary developmental phenotype caused by the knock-out of these genes [22]. This could indicate that *Pkd1* zebrafish morphants or mutants might not be very adequate models for ADPKD as a kidney disease.

### 3.4. Rodent Models

As small mammals, the mouse and the rat present several advantages for biomedical research, that include the fact that their physiological and genetic structures are closer to those of humans than the aforementioned models, together with a relatively short life cycle and affordable housing costs [144]. In addition, in particular thanks to the generalization of the CRISPR/Cas9 genetic editing system, the generation of specific transgenic mouse strains has been significantly facilitated and accelerated. Two useful resources to gather information about specific murine models are the interconnected databases MouseMine (http://www.mousemine.org/mousemine/begin.do) and Mouse Genome Informatics (MGI (Mouse Genome Informatics, a resource for laboratory mouse hosted by the Jackson Laboratory, http://www.informatics.jax.org/) [145]. A large number of murine models are commercially available, and distributers can easily be found on the website of the “International Mouse Strain Resource” (IMSR, http://www.findmice.org/). Even though transgenesis is more complicated in the rat than in the mouse, technical advances have recently been made and, as a result, the number of transgenic rat models for human disease will certainly significantly increase in the coming years [146].

**Figure 3:**
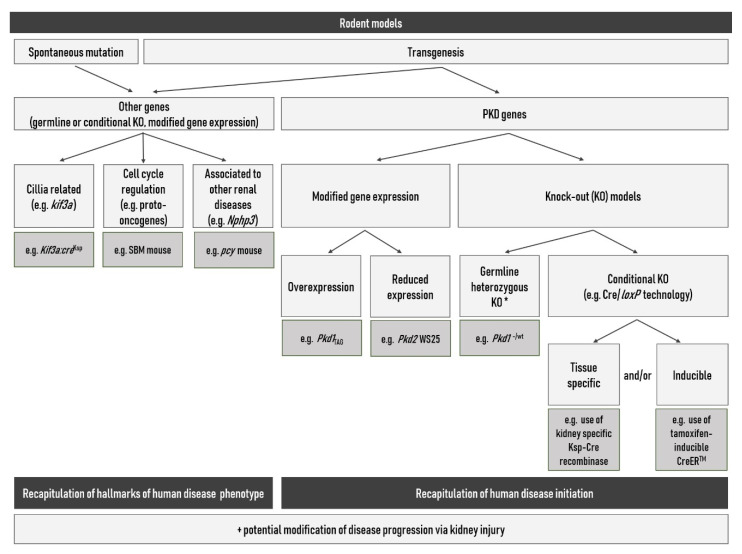
Schematic overview of the different types of rodent ADPKD models (adapted from [28]). *: homozygous germline knock-out embryonically lethal.

Hence, numerous rodent models have been developed for the study of ADPKD (Figure 3). These models can roughly be divided in two categories: those arising from spontaneous genetic mutations and those produced through genetic engineering [28]. In addition, there are models recapitulating human disease initiation via modified expression (hypomorphic alleles, conventional heterozygous knock-out alleles, conditional and potentially inducible knock-out alleles, overexpression) of either the human or the rodent PKD genes on the one hand and, on the other hand, models recapitulating more or less precisely an ADPKD phenotype due to mutations in various other gene families (e.g., cell cycle, developmental regulation, cilia) (Table 3). While the second type of models is certainly important to reveal underlying mechanisms and regulatory pathways involved in some aspects of the pathological phenotype, and therefore potential modifier genes, their use in drug discovery is debatable as they do not mimic human disease initiation [147]. For more details, the reader is referred to several excellent and comprehensive reviews on rodent models used in PKD research that have been published over the last two decades [28,147,148,149,150,151,152]. The objective of the present review is not to provide an exhaustive list of all existing models but rather to give a broad overview of the various types of models that can be used to study ADPKD. Thus, in this paragraph, we will briefly introduce only the rodent models that seem to us most closely related to human ADPKD.

Even though rodent models based on the engineering of *PKD1* and *PKD2* genes seem to be an obvious choice to study ADPKD pathophysiological mechanisms and for drug screening, these models present several limitations. First, as supposed to be the case in humans, homozygous loss of function of the murine *Pkd1* or *Pkd2* genes by conventional knock-out is embryonically lethal, whereas animals heterozygous for those mutations only develop very mild cystic disease. In humans, the pathology slowly progresses over decades before it leads to end-stage renal disease, a feature that fits with the ‘two-hit’ hypothesis that postulates that cyst formation is initiated when a somatic mutation occurs in the wild-type allele of the *PKD* gene [169]. Hence, according to this hypothesis, the lifespan of the heterozygous *Pkd* knock-out murine models might simply be too short to effectively recapitulate human ADPKD. A murine model supporting this hypothesis is the *Pkd2* WS25 model in which a mutant exon 1 has been introduced in tandem with the wild-type exon 1 in the *Pdk2* locus, resulting in an unstable allele [170]. This allele is prone to homologous recombination resulting either in the reversion to the wild-type allele or a true null mutant allele of *Pkd2*. When the unstable WS25 allele is combined with a true null allele of *Pkd2* (*Pkd2*^WS25/-^), animals escape embryonic lethality but show decreased survival with a median age at death of 65 weeks. Furthermore, *Pkd2*^WS25/-^ mice develop bilateral kidney cysts as well as liver cysts in a variable manner at 10–11 weeks of age and, in some animals, pancreatic cysts could be observed at 3 months of age [170,171]. However, the ‘two-hit’ hypothesis for cyst initiation has today been expanded to a gene dosage hypothesis where the level of functional polycystin proteins play a crucial role for cyst formation. It follows that, if the gene dosage hypothesis does not exclude a ‘second hit’ via somatic mutations, it further includes other factors that could modify *PKD* expression [18,27]. This hypothesis is supported by animal models displaying reduced or increased expression of the *Pkd* genes. A mouse model carrying a hypomorphic *Pkd1* allele induced by aberrant splicing of intron 1, thereby reducing the quantity of functional polycystin-1 to 13–20%, was presented by Leeuwen et al. in 2004 [172]. In this so-called *Pkd1*^nl^ model, overall survival was decreased but disease progression was very variable. Animals showed bilateral kidney cysts, mild cystic disease in the liver and pancreas, and development of aortic aneurysms, thus exhibiting a large spectrum of the human ADPKD symptoms. Furthermore, in 2012 Katharina Hopp et al. described a new murine model carrying an hypomorphic *Pkd1* allele, the *Pkd1^RC/RC^* mouse [173]. This model was developed through the knock-in of a clinically relevant and incompletely penetrant *PKD1* allele (p.R3277C). This particular allele was first identified in a consanguineous family and, of note, homozygous carriers of this allele are viable and present mild to typical cystic disease, supporting the gene dosage hypothesis. *Pkd1^RC/RC^* mice develop a slowly progressing polycystic kidney disease with embryonic cyst initiation, present cystic lesions in the liver and elongated primary cilia in collecting ducts, while heterozygous *Pkd1^RC^*^/null^ show a more severe disease progression and significantly reduced survival when compared to *Pkd1^RC/RC^* homozygous animals. The slowly progressive nature of the disease in this model, as well as the clinical relevance of the introduced mutation make it particularly interesting for the study of ADPKD. Its validity as a preclinical model for ADPKD drug efficiency studies was further established by the observation of a positive effect of tolvaptan in this model. This model also served to show the activity of pasireotide, a synthetic analogue of somatostatine which, as tolvaptan, indirectly reduces adenylyl cyclase 6 (AC6) activity and has hence proven effective in slowing cyst progression. In this study, pasireotide or tolvaptan alone were active, and their combination led to an additive effect [174]. A model in which *Pkd1* is overexpressed, the *Pkd1*_TAG_ mouse, was presented in an elegant study by Kurbegovic et al. in 2010 [175]. They injected a *Pkd1*_TAG_ construct (murine *Pkd1* carrying a silent point mutation in order to distinguish it from the endogenous gene) in murine oocytes and analyzed three of the resulting mouse lines carrying either 2, 6, or 15 copies of the transgene. They observed a copy number-dependent increase of both *Pkd1* mRNA level and polycystin-1 expression. Furthermore, all hallmark symptoms of human ADPKD were evaluated in this study: *Pkd1*_TAG_ mice present a decreased lifespan presumably due to renal failure, develop kidney as well as hepatic cysts, cardiac abnormalities and, in some cases, increased blood pressure was observed. Importantly, the severity of the ADPKD disease phenotype developed was shown to be dependent on the number of copies of the transgene integrated in the genome, with the *Pkd1*_TAG_ line 26 (*Pkd1*_TAG_26, bearing 15 copies of the transgene) presenting the most severe phenotype and the shortest lifespan. This model somewhat supports and expands the gene dosage hypothesis through the experimental validation that a dysregulation of polycystin-1 through overexpression of the protein can induce an ADPKD specific phenotype in rodents, in addition to significantly reduced polycystin-1 expression as it has previously been shown. This is further supported by the observation of an overexpression of polycystin-1 in end stage ADPKD renal cystic epithelia, even though it has been suggested that this constitutes only a secondary phenomenon reflecting the differentiation stage of end-stage cystic epithelia, thus being only a consequence of the mechanisms initially triggering cyst development [176]. 

The development of conditional knock-out models has also enabled prevention of the embryonic lethality of homozygous null mutations of the *Pkd* genes displayed by conventional germline knock-out mice. The widely employed Cre/*loxP* technology permits to control transgene expression in a tissue specific or inducible manner [177]. Briefly, two specific, short nucleotide sequences, the *loxP* sites, are introduced in either side of the target gene (or of part of it) in such a manner that they do not interfere with normal gene expression. To modify the expression of the so-called “floxed” (flanked by *loxP* sites) gene, another transgene, the *cre* gene, must be present in the genome. *Cre* encodes a recombinase, which specifically recognizes the *loxP* sites and can excise any nucleotide sequence comprised between two of those sites. If *cre* expression is controlled by a tissue-specific promoter, inactivation of the floxed gene only takes place in tissues where the recombinase is expressed. One example of such a strain used in ADPKD research is the *Pkd1*^flox^:Ksp-Cre model presented in 2008 by Shibazaki et al. [178]. In this model, exons 2 to 4 of the *Pkd1* gene are floxed and recombinase expression is restricted to renal tubules via the Ksp-cadherin promotor [179], permitting kidney specific inactivation of *Pkd1*. *Pkd1*^flox^:Ksp-Cre animals show a rapid progression of polycystic kidney disease and die, between the postnatal days 14 and 17, presumably due to kidney failure. 

The availability of systems in which recombinase activity is inducible by the administration of a specific substance to the animals, for example tamoxifen, permits to produce animals presenting a ubiquitous *Pkd* knock-out while avoiding embryonic lethality. In 2007 Piontek et al. used the CAGGCre-ER^TM^ strain (Jackson Laboratory stock number 004682) in which the recombinase expression is induced by tamoxifen, in combination with a floxed *Pkd1* allele and demonstrated that the timing of *Pkd1* disruption had a significant influence on disease severity and progression [180]. After tamoxifen induced *Pkd1* knock-out, all the animals develop renal as well as hepatic cysts, thus presenting two hallmark features of ADPKD. In addition, disease progression was observed to be strikingly faster when tamoxifen was administrated before postnatal day 13.

Today, numerous Cre strains carrying *cre* alleles with tissue-specific and/or inducible recombinase expression have been created in various genetic backgrounds and a large number are commercially available (Cre strains and corresponding expression data and references can be found on the MGI website, an international database resource for laboratory mouse hosted by the Jackson Laboratory, http://www.informatics.jax.org/home/recombinase). In combination with strains carrying floxed alleles of ADPKD relevant genes, these various Cre strains allow the creation of a large number of models to study specific features of ADPKD pathology and to elucidate the underlying molecular pathways and their timing. As it would exceed the scope of this broad review to describe the specific transgenic *Pkd1* and *Pkd2* murine models, here again we refer our reader to the recently published chapter describing those models in detail, as well as the divers techniques used to assess the PKD phenotype in those animals, in the book *Polycystic Kidney Disease* edited by Jinghua Hu and Yong Yu [181].

In addition to these models created by targeting the orthologues of the human *PKD* genes, rodent models displaying phenotypical similarity to ADPKD pathology resulting from mutations in other genes have been used in ADPKD research. A brief summary of the most frequently employed models is presented in Table 3. These models have helped to gain important insights in the molecular pathways and gene networks relevant to polycystic kidney disease. For example, the SBM mouse developed by Trudel et al. in 1991 overexpresses the proto-oncogene *c-myc* predominantly in the kidney and develops a renal phenotype similar to ADPKD with kidneys being the sole or predominantly affected organ [156,157]. Thus, the SBM murine model underlines that the dysregulation of tubular epithelial cell proliferation and apoptosis is sufficient to induce renal cyst formation and further supports the idea that elevated *c-myc* expression plays a major role in the pathogenesis of human PKD. Lin et al. demonstrated that kidney specific inactivation of the KIF3A subunit of kinesin-II, a protein essential to cilia formation, results in large cystic kidneys by postnatal day 28 (P28) and the complete replacement of renal parenchyma by large cysts by P35 [155]. This study thus strengthens the hypothesis that ciliary defects play a crucial role in PKD disease development. It is worth noting that a number of other rodent models developing renal cysts and other symptoms similar to ADPKD exist, in which dysregulation of cilia-associated proteins, proto-oncogene expression, or the mutation of genes related to other human diseases (e.g., *Pkdh1* in the Pck rat, autosomal recessive polcystic kidney disease, ARPKD) are responsible for the observed phenotypes [151,152,182]. This suggests that common molecular pathways are causative for renal cyst formation and that the polycystin proteins participate, directly or indirectly, to various major regulatory networks implicated, among others, in cell proliferation and apoptosis [150]. 

Importantly, in view of the large number of PKD rodent models available, of which each presents some specific features, advantages and limitations, Hester Happé and Dorien J. M. Peters proposed, in their review of animal models used in ADPKD research, that at least two different models should be used in preclinical testing for potential therapies [28]. This point of view is strongly supported by the fact that different therapeutic interventions do not display the same efficiency in different rodent models of PKD [150].

### 3.5. Other Mammalian Models 

In Persian cats, a spontaneous mutation arose, which is inherited in an autosomal dominant pattern and causes an adult onset ADPKD phenotype, with progressive renal cyst formation leading to renal failure later in live (>7 years), biliary fibrosis and hyperplasia, sporadic hepatic cysts and slightly increased blood pressure [183,184,185]. The gene responsible for ADPKD in Persian cats is the feline *PKD1* gene, and a C to A transversion resulting in a premature stop codon inducing the loss of ~25% of the protein’s C-terminus was identified in affected cats by Lyons et al. [186]. Even though this model is rarely used in ADPKD research, its striking genetic and phenotypic similarities to human ADPKD together with its significantly longer lifespan than rodent models make it an attractive tool to study ADPKD disease progression and to test therapeutic approaches. In 2019, in an elegant study, Torres et al. presented the therapeutic potential of a ketogenic diet which was evaluated in two rodent and the feline models of ADPKD [187]. The results of their study suggest that a high-fat, very-low-carbohydrate diet might significantly slow down disease progression without any pharmacological intervention, thereby representing a promising therapeutic approach which deserves further investigations.

Porcine species are also increasingly gaining attention as animal models of human disease in biomedical research and the remarkable physiological and functional resemblance between porcine and human kidneys make swine very good models for human kidney diseases [188,189]. In addition, physiological and structural similarities between human and porcine organs make this species the most likely candidate for the production of organs intended for human organ replacement therapy. In line, the possibility of xenotransplantion of organs from pig into human is currently investigated as a possible solution to organ shortage [134]. In 2011, a research team of the State Key Laboratory for Agrobiotechnology at the China Agricultural University of Beijing directed by Ning Li characterized the porcine *PKD1* and *PKD2* genes. They found strong sequence similarities between the porcine and human *PKD* genes and proteins, as well as in their expression patterns [190,191]. In 2015, the same research team reported that a mini-pig model of ADPKD had been developed by mono-allelic knock-out of the *PKD1* gene [192]. In this model, *PKD1* expression was reduced at a transcriptional and translation level, macroscopically visible kidney cysts were detectable from 5 months of age and by 24 months of age these cysts began to deform the normal kidney shape. Furthermore, liver cysts were reported to be present in all investigated animals. This model was first used in a study by Lian et al. published in 2019 [193], in which the effect of single and combined treatments with 2Deoxy-D-glucose (2DG, an inhibitor of glycolysis reported to reduce cysts growth in mouse PKD models [194,195]) and metformin (an AMP-activated protein kinase inhibitor) on ADPKD progression was investigated. They found that all drug treatments significantly inhibited cyst growth in the ADPKD mini-pig model, the combined therapy being the most effective. Furthermore, none of the treatments led to apparent side effects. The research team from the China Agricultural University of Beijing also developed two further potential transgenic mini-pig models of ADPKD, one via the induction of *c-myc* overexpression and the other via the induction of *PKD2* overexpression [196,197]. However, no kidney cysts or signs of impairment of renal function were observed in these models after 6 months and 12 months respectively. The authors suggested that the lack of symptoms might be due to the slowly progressing nature of ADPKD pathology and stated that both models continued to be observed. However, no update has been published since the original publications from 2013.

## 4. Discussion

This current review illustrates the vast number of tools available today for the study of ADPKD, as well as some promising emerging technologies, like the ‘kidney-on-a-chip’. This large choice can, however, be confusing and, since no perfect model of ADPKD exists to date, the suitability of a chosen model in view of a specific research hypothesis must be thoroughly considered. This issue is currently partly addressed by the multimodal approach most research teams choose. Importantly, the choice of such an approach is the result of a balance between a rigorous scientific strategy, with the final aim to validate results in several distinct experimental conditions, ethical concerns, as well as economic considerations, not to mention the need to obtain publishable results in a reasonable timeframe. Most in vitro techniques present the advantages of being relatively rapid, cost-effective, and independent from any ethical issues (as compared to in vivo models). Hence, a large number of reproducible results can be rapidly obtained through standardized protocols and manipulation techniques. Even though the ‘kidney-on-a-chip’ might represent an attractive solution to a certain extent, in vitro models in use today are studied by essence in a complete absence of any physiological context. This may be viewed as an advantage for the investigation of isolated cellular mechanisms, but those models cannot give any indications on complex in vivo tissue or organ interactions, compound metabolization or potential off-target in vivo toxicity of a parent compound or of its metabolites. Small and widely-used invertebrate models, like the worm *Caenorhabditis elegans*, and non-mammalian vertebrate models, like the zebrafish *Danio rerio*, may be considered as they represent an interesting alternative and compromise between in vitro models and complex in vivo models [85,87]. Indeed, their housing costs are relatively low, their numerous, and by definition ex utero developing embryos are, up to a certain developmental state in the case of vertebrates, of no concern to the legislation on the protection of animals used for scientific purposes [86]. In addition, as the embryos and larvae of those animals are often transparent, they allow direct observation of physiological processes and facilitate the development of high-throughput technologies to test the toxicity or efficacy of a large number of different compounds. Furthermore, an increasing number of tools for the genetic manipulation of such organisms are available as of today. The genomes of these small model organisms have been sequenced, which allows the study of protein expression and function in a physiological context and thereby to create human disease models in these very distant relative species. Especially in biomedical research, high-throughput screening using in vitro or small in vivo models is often the first step to identify potential therapeutic molecules. For ADPKD research, the high-throughput platforms using 3D cysts [66] or kidney organoids derived from *PKD* knock-out hPS cells [36], as well as the automated imaging pipeline using zebrafish larvae [143] in combination with *Pkd1a/b* or *Pkd2* morpholino oligos could be particularly useful to detect promising candidates while limiting false positives. The efficiency and lack of toxicity of the potential therapeutic molecules identified in such a way must then be validated in animal models more closely related to humans, such as the commonly used rodent models. Those species have again the advantage that housing costs are relatively low compared to larger mammals such as dogs or mini-pigs, while producing a relatively high number of offspring [144]. Furthermore, those organisms are very well studied and the scientific community has a large number of different transgenic or non-transgenic strains readily available. However, even though rodents are more closely related to humans than the aforementioned organisms and the fact that a considerable number of transgenic mouse models of human diseases exists today, results obtained in those models are not always fully transposable to humans. Even though murine models have proven reliable models of aging, in ADPKD research notably, the short lifespan of those animals might be a limiting factor. The majority of human ADPKD patients present a heterozygous mutation in one of the *PKD* genes and the disease progresses over decades before renal function is impaired to a point that renal replacement therapy is necessary; while in murine models, heterozygous *Pkd* mutations—which better recapitulate the human disease—cause a mild and very variable disease phenotype. Somatic mutations of the second non-mutated *PKD* allele are supposed to play a role in the initiation of cyst formation in ADPKD patients, and even though somatic mutation rates may differ between organisms, the short lifespan of rodents might thus explain the only very mild phenotype observed as a consequence of heterozygous *Pkd* mutations [18,198]. In this sense, the ADPKD cat and mini-pig models seem to recapitulate human ADPKD pathogenesis more accurately than rodent models. Porcine models of human disease seem especially promising in view of their strong physiological similarity to humans. However, up to now, only few research facilities have the required equipment, staff experienced in handling, and an adequate husbandry to house those species, not to forget that the housing costs of such models are high. However, in the long term, increasing use of specific mini-pig models of human disease in biomedical research might eventually decrease these expenses. In addition, the significant financial loss due to the clinical trials of molecules which seemed effective and promising in preclinical rodent models, but that later turned to be ineffective or to present important adverse effects in humans may also be in favor of the use of cat and mini-pig models for ADPKD. Example of such molecules that showed promising results in preclinical studies carried out on rodent models of PKD are the mTOR inhibitors sirolimus (rapamycin) and everolimus. While both molecules efficiently slowed down disease progression in the Han:SPRD cy/+ rat [199,200,201] and rapamycin further proved to be efficient in the *bpk* mouse and the *orpk*-rescue mouse [202], no beneficial effect of rapamycin or everolimus treatment could be observed in clinical trials with ADPKD patients [203,204]. In this context, it may be interesting to note that none of the PKD rodent models employed during the preclinical evaluation of those compounds was based on mutations in the *Pkd* genes.

Finally, we share the view of Hester Happé and Dorien J. M. Peters [28] that ADPKD treatment strategies should be tested in at least two different animal models, of which at least one should be based on a *PKD* gene mutation. Vasopressin V_2_ receptor antagonists OPC-31260 and its derivative tolvaptan (OPC-41061) have for example shown effectiveness in several distinct rodent models of PKD, among those the PCK rat, the *pcy* mouse, the *Pkd2* WS25 mouse, and a conditional kidney specific *Pkd1* knock-out model [205,206,207,208], as well as in the *cpk* mouse [209], a model of autosomal recessive PKD (ARPKD), before and while clinical testing for its effectiveness in ADPKD treatment was conducted. We would also argue in favor of the integration in studies investigating potential disease treatments of larger mammals like the ADPKD cat or the recently developed ADPKD transgenic mini-pig. However, before tests are carried out in animals, potential therapeutic compounds have to be identified in adequate in vitro models in order to limit the wasteful use of laboratory animals. In ADPKD research, transgenic hPSC-derived kidney organoids are potentially the most promising in vitro model available today. A second validation of identified candidates might then be performed in zebrafish models of ADPKD induced by *Pkd2* or *Pkd1a/b* morpholino injection before mammalian models are employed. In any case, the choice of specific models must be anticipated according to the chosen strategy and we hope that this review will provide some helpful information for this process of decision-making. Finally, the InterMOD project (MOD: Model Organism Database), which “aims to make it easier to carry out cross-MOD comparative analysis and to relate MOD data to medical research” [210] should also be mentioned in this place. Different MODs, among those FlyMine (https://www.flymine.org/flymine/begin.do), ZebrafishMine (http://www.zebrafishmine.org/begin.do), MouseMine (http://www.mousemine.org/mousemine/begin.do), RatMine (http://ratmine.mcw.edu/ratmine/begin.do) and HumanMine (https://www.humanmine.org/humanmine/begin.do), participate in the project and thus permit cross-species analysis of biological data. Therefore, this project constitutes a helpful and interesting tool for the integration and translation of model organism research into medical applications, as well as for the comparison of model organisms in view of a specific research project.

## 5. Conclusions

The existence of such numerous tools and models to conduct ADPKD research results from the rigorous and important research that has been carried throughout the last decades. The valuable findings which have been collected through the use of those tools help to better understand this complex disease and have made the discovery of the first drug treatment permitting to slow down ADPKD progression possible. Furthermore, the existence of several in vitro and in vivo models for this human disease provides researchers with the flexibility to choose among the diverse models those which best suit their specific research questions in order to obtain more reliable and reproducible results. However, as highlighted in this article, as of today no model perfectly recapitulates the human disease and thus special attention has to be paid to the specific characteristics of each model when establishing a research protocol. We therefore hope that this review will provide ADPKD researchers a comprehensive overview of the existing and emerging models used to study this disease, together with their main advantages and limitations.

## Figures and Tables

**Table 1:** Broad overview of the advantages and limitations of the different in vitro models used in ADPKD research

	Advantages	Limitations	Ref.
**Cell Type**			
Primary cells	Cost-effective Standardized protocols No genetic modification potentially causing phenotypic changes Controlled environment and simplicity of the system remove confounding factors and facilitate the interpretation of results	Limited viability permits only the evaluation of acute responses Necessity to obtain new isolates frequently, which leads to differences in genetic background and may confound comparability and reproducibility of results Potential ethical concerns about the availability of tissue samples Mechanical stress during cell isolation Inadvertent selection of wrong cell types	[33,34]
Immortalized cell lines	Cost-effective Standardized protocols Numerous resources Stability of genetic background increases reproducibility Extended culture possible Controlled environment and simplicity of the system remove confounding factors and facilitate the interpretation of results	Genetic changes resulting from immortalization can cause phenotypic changes as compared to primary cells Long-term cell passing can equally cause genotypic and phenotypic variation which can cause heterogeneity in cultures Risk of (undetected) contamination with other cell lines and mycoplasma	[33,34,35]
Pluripotent stem cells (PSC)	Patient derived PSCs permit to study human disease in relevant in vitro models Possibility of generating genetically-modified PSCs enabling the production of organoids modeling human disease Permit the differentiation of distinct cell types and the production of organoids Induced pluripotent stem cells limit ethical concerns	Not all cell types are available (e.g., proximal tubule cells are the only immortalized renal epithelial cell lines available as of today) Variability of genetic background in patient-derived PCSs Ethical concerns relative to embryonic pluripotent stem cells More tedious and costly as compared to primary or immortalized cell culture	[32,33,34,36,37]
**Culture System**			
2D cell culture	Simple, reproducible, and cost-efficient Traceable and controlled environment facilitating the unravelling of cellular processes and the interpretation of results High throughput applicability	Monolayer culture of single cell types is not representative of the complexity of an organ Lack of the physiological environment defining and influencing cellular functions	[34,38]
Spheroid culture (3D cysts)	Closer to living tissues than 2D culture (biostructural and biofunctional properties) Relatively simple, reproducible, and cost-effective Traceable and controlled environment facilitating the unravelling of cellular processes and the interpretation of results High throughput applicability	Spheroid structure may cause difficulties to observe pathological cyst structures Culture of single cell types is not representative of the complexity of an organ Lack of the physiological environment defining and modifying cellular functions	[38,39]
Embryonic kidney culture (rodents)	Presence of different cell types and organ structures like the nephron Embryonic kidney culture from well characterized transgenic disease models can provide valuable insights in disease mechanisms as a maximum of factors can be controlled (e.g., animal husbandry environment, cell culture conditions) and results can be related to specific in vivo phenotypes	Only representative of an early developmental stage, which can limit the reliability of obtained results, notably in the case of late-onset genetic diseases Same ethical concerns as for animal experimentation, thus also potentially limiting high throughput approaches Lack of standardized protocols Limited viability (4–10 days) Lack of physiological environment defining and influencing cellular functions Tedious and costly as compared to 2D and 3D culture systems	[40,41]
Kidney organoids	Organoids can be established from genetically modified or patient-derived PSC Most representative in vitro model of complex organs available to date (containing nephron-like epithelial structures, including podocytes and tubular segments) High throughput applicability Traceable and controlled environment facilitating the unravelling of cellular processes and the interpretation of results	Representative of a nascent developmental phenotype Lack of vasculature and of the organ specific physiological environment (e.g., fluid flow) Possible lack of reproducibility for organoids derived from patient PSCs Limited viability (<1 week) which restricts the ability to mimic the development of chronic diseases Tedious and costly as compared to other culture systems	[32,33,36,37]
**Origin of Cells**			
Human	Culture of cells extracted from a diseased organ is possible and permits the study of particular pathological cellular mechanisms Interspecific differences in cell or organ functions do not need to be considered	ADPKD patient specific primary cells are generally representative of an advanced disease stage and no control cells with an identical genetic background are available Differences in genetic background can complicate the reproducibility and interpretation of results Possible ethical concern depending on the cell type (e.g., embryonic stem cells)	[33,34,39]
Animal	The culture of cells extracted from well characterized transgenic disease models can provide valuable insights in disease mechanisms as a maximum of factors can be controlled (e.g., animal husbandry environment, cell culture conditions) and results can be related to specific in vivo phenotypes Lesser ethical concern than for cells of human origin	The phenotypes displayed in culture may differ from those of human cells	[34,39]

**Table 2:** Overview of the advantages and limitations of invertebrate and lower vertebrate ADPKD models.

Species	Excretory System	Advantages	Limitations	Applications (ADPKD)	Ref.
**Invertebrates**					
***C. elegans***	Rudimentary kidney-like organ consisting of a single cell	LOV-1 and PKD-2 present a high structural similarity to human polycystins Simple assessment of cilia integrity and phenotypic readouts of cilia dysfunction Very low maintenance cost (petri dishes, liquid culture, or fermenter-like devices for mass culture), high number of offspring (hermaphrodites: ~300, fertilized males ~1000) Very fast generation time (fertilization to hatching: 14 h; post-embryonic development through four larval stages: 35 h, lifespan: 2–3 weeks) Cryoconservation possible High throughput applicability Immotile cilia are not required for normal development or viability (knock-outs of cilia genes do not cause lethality) Comparatively simple and cost-effective genetic manipulations (RNAi screening, CRISPR/Cas9, …) and fast and simple generation of double, triple or n-ple mutants In vivo analysis of cell morphology, microarchitecture and protein sublocalization (*C. elegans* are transparent)	*lov-1* and *pkd-2* mutations induce impaired male mating behavior without apparent malformations or embryonic lethality contrary to the situation in humans Limited usefulness for ADPKD drug screening Lack of a multicellular excretory system	Research on cilia function and gene networks (implications for ciliopathies like ADPKD) Polycystin function in extracellular vesicles Unravelling of in vivo drug action by various chemobiological approaches (bridging the gap between in vitro and mammalian in vivo drug action and toxicity)	[85,92,101,131,132]
***D. melanogaster***	Aglomerular, Malpighian tubules (analog to renal tubules) and nephrocytes (analog to podocytes)	*BicC* mutants develop a renal cystic phenotype which improves upon rapamycin or Smac treatment, which suggests pathways similar to those of rodent ADPKD models *Amo* (*PKD2* homologue) mutants present no lethality but impaired male mating behavior Low maintenance cost Fast generation time (fertilization to adult (embryogenesis + three larval stages + pupation): 10 days, lifespan: 40–50 days) High throughput applicability Comparatively simple and cost-effective genetic manipulations (RNAi screening, CRISPR/Cas9,…)	Lack of a *PKD1* homologue Amo lacks the C-terminal tail Lack of ciliated epithelia Evolutionary distance to humans Lack of ‘archiving’ techniques, permanent maintenance of mutant strains necessary Important differences in metabolism between humans and flies which can limit the relevance of their use for drug screening	Drug screening Deciphering and modeling molecular pathological mechanisms of PKD	[84,85,133,134,135]
**Vertebrates**					
***D. rerio***	Pronephros with two nephrons (embryos), mesonephros (adults)	Several models developing kidney cyst have been developed, among those *Pkd2* morphants and mutants as well as *Pkd1 a* or *Pkd1 a/b* morphants Simple phenotype readouts for *Pkd* mutants/morphants through body/tail curvature *Pkd1 a* mutants present liver cysts Simple analysis of kidney function possible via dye clearance assay Cryoconservation of sperm possible High number of offspring (~400) by spawning High throughput applicability (early developmental stages) Comparatively simple and cost-effective genetic manipulations (RNAi screening, CRISPR/Cas9,…) and possible transient gene knock-out via morpholino injection, Observation of embryonic development processes and in vivo live imaging techniques (transparent embryos with an ex utero development)	Two paralogs of the *Pkd1* gene (*Pkd1 a* and *Pkd1 b*) with distinct expression patterns Variability of the phenotype developed by *Pkd* morphants and/or mutants Only embryonic developmental stages can be used for high throughput drug screening whereas ADPKD is a late-onset and slowly progressing disease Motile cilia in the pronephros Maintenance requires centralized water tanks with controlled temperature and light/dark cycle High throughput screening limited to embryonic stages Morpholino phenotypes should phenocopy the mutant phenotype and be validated via the rescue of the phenotype through injection of the correspondent mRNA Paleotetraploid genome (potentially complicating genetic analysis and mutant production), Embryos are protected by the chorion during the first 48–72 h post fertilization which can impair drug penetrance, whole mount immunostaining and in situ hybridization procedures; the removal of the chorion (pronase treatment) fragilizes the embryos (necessity to avoid contact with air or plastic)	Drug screening Molecular pathological mechanisms of PKD	[22,121,123,128,130,136,137,138]
***Xenopus*** ***(X. laevis*** **and** ***X. tropicalis)***	Pronephros with two nephrons (embryos), mesonephros (adults)	Several morphants (*Pkd1*, *Pkd2*, *Bicc*, *Gnas*) developing a PKD phenotype have been described Long lifespan and fertility (about 10 years) which facilitates the maintenance of transgenic strains and the observation of late onset diseases in mutants (in comparison to invertebrate, lower vertebrate, as well as rodent models) Simple analysis of kidney function via dye clearance assay possible Cryoconservation of sperm possible Very high number of offspring (~1000 eggs/female) and in vitro fertilization permits production of a large number of synchronized embryos High throughput applicability (early developmental stages) Comparatively simple and cost-effective genetic manipulation (RNAi screening, CRISPR/Cas9,…) and transient gene knock-out via morpholino injection possible The only tetrapods with free-living embryos, highest order permitting high throughput procedures Observation of embryonic development processes and in vivo live imaging techniques (transparent larva and tadpoles with an ex utero development) Larger and more robust embryos than zebrafish permitting microsurgery and unilateral tissue specific injections which permits the production of targeted and tissue specific knock-down or knock-out models (with the non-injected side as an internal control) *X. tropicalis* presents a true diploid genome Drug screening results comparable to those carried out in zebrafish	Only embryonic developmental stages can be used for high throughput drug screening whereas ADPKD is a late-onset and slowly progressing disease Motile cilia in the pronephros Lower availability of resources and technical tools than for zebrafish (notably concerning ADPKD relevant models) High throughput screening limited to embryonic stages (stage 43 of embryonic development corresponds approximatively to embryonic day 18.5 in mice) Morpholino phenotypes should phenocopy the mutant phenotype and be validated via the rescue of the phenotype through injection of the correspondent mRNA *X. laevis* presents a paleotetraploid genome (potentially complicating genetic analysis and mutant production),	Drug screening Molecular pathological mechanisms of PKD	[87,139]

**Table 3:** Examples of rodent models frequently employed in ADPKD research, in which a phenotype closely resembling ADPKD is caused by mutations in genes other than the *PKD* genes. Models developing a phenotype rather resembling ARPKD than ADPKD, such as the *bpk* mouse in which the polycystic phenotype is caused, as in the *jcpk* model, by a mutation in the *Bicc1* gene [153] or the *orpk* mouse (oak ridge polycystic kidney) carrying a hypomorphic allele of *Ift88* [148,154] have not been included in this table. P = postnatal day.

	Example	Gene	Construct/Mutation	Human Orthologue	Protein	Phenotype	Ref.
Induced mutation (cilia associated gene)	*Kif3a*:*cre^Ksp^*	*kif3a*	Kidney specific inactivation	*KIF3A*	KIF3A (subunit of kinesin-II)	Renal parenchyma replaced with cysts by postnatal day 35	[155]
Induced mutation (proto-onco gene)	SBM mouse	*c-myc*	*c-myc* overexpression via the introduction of a transgene (*c-myc* coding region under the control of the SV40 enhancer and β-globin promoter, different lines with variable copy number of the transgene)	*MIC*	Myc proto-ongogene protein	Progressive polycystic kidney disease with atypical plasmacytic infiltrates, anemia, premature death due to renal failure at 2 weeks to 4 months of age	[156,157]
Spontaneous mutation	*jcpk* mouse	*Bicc1*	Single base-pair change (AG→AA) in the splice acceptor site of exon 3 causing a frameshift resulting in a premature stop codon	*BICC1*	Bicc1	Progressive polycystic kidney disease, hepatic and pancreatic dilated ducts, gall bladder enlargement, premature death between P7-P10	[153,158]
	*jck* mouse	*Nek8*	Nucleotide substitution (G→T) resulting in an amino acid change (V→G)	*NEK8*	Nek8 (NIMA (never in mitosis-A) related kinase)	Slowly progressive polycystic kidney disease, decreased fertility in males from 15 weeks of age, premature death at 20 to 25 weeks of age	[159,160]
	*kat* and *kat^2J^* mouse	*Nek1*	*kat*: internal deletion *kat2J:* single base-pair insertion causing a frame shift resulting in a premature stop codon	*NEK1*	Nek1 (NIMA (never in mitosis-A) related kinase)	Late onset, slow-progressing polycystic kidney disease, facial dysmorphism, dwarfing, male sterility, anemia, and cystic choroid plexus, premature death either before weaning or at 1 year of age (faster disease progression in *kat^2J^*)	[161,162]
	*pcy* mouse	*Nphp3*	Amino acid substitution (I→S)	*NPHP3* (associated with nephronoph-thisis)	Nephrocystin-3	Slow progressive polycystic kidney disease with cerebral aneurysms, premature death and chronic inflammatory infiltrates in advanced stages, with a mean age at death of 6.5 months (females) and 8.2 months (males)	[163,164]
	Han:SPRD cy/+ rat (Cy-rat)	*Anks6* (*Pkdr1*)	Missense point mutation resulting in an amino acid substitution (R→W)	*ANKS6*	Ankyrin repeat and SAM domain-containing protein 6	Slowly progressing renal enlargement by cyst formation, phenotype more severe in males than in females, premature death (males from 6 months of age, later for females)	[165,166,167,168]

## Abbreviations

2/3D	2/3-dimensional
2DG	2Deoxy-d-glucose
ADPKD	Autosomal dominant polycystic kidney disease
ARPKD	Autosomal recessive polycystic kidney disease
CFTR	Cystic fibrosis transmembrane conductance regulator
ECM	Extracellular matrix
ESC	Embryonic stem cells
ESRD	End-stage renal disease
*Gnas*	Guanine nucleotide binding protein G subunit α isoform (short)
hESC	Human embryonic stem cell
hPSC	Human pluripotent stem cell
iPSC	Induced pluripotent stem cell
ISMR	International Mouse Strain Resource
MDCK	Madin–Darby canine kidney
PC-1/2	Polycystin 1/2
PKD	Polycystic kidney disease
PPE1A	Phosphodiesterase 1A
